# Synergetic effect of green synthesized reduced graphene oxide and nano-zero valent iron composite for the removal of doxycycline antibiotic from water

**DOI:** 10.1038/s41598-022-23684-x

**Published:** 2022-11-12

**Authors:** Ahmed M. Abdelfatah, Nourhan El-Maghrabi, Alaa El Din Mahmoud, Manal Fawzy

**Affiliations:** 1 Environmental Sciences Department, Faculty of Science, Alexandria University, Alexandria, 21511 Egypt; 2 Green Technology Group, Faculty of Science, Alexandria University, Alexandria, 21511 Egypt; 3 National Biotechnology Network of Expertise (NBNE), Academy of Scientific Research and Technology (ASRT), Cairo, Egypt

**Keywords:** Environmental sciences, Materials science

## Abstract

In this work, the synthesis of an rGO/nZVI composite was achieved for the first time using a simple and green procedure via* Atriplex halimus* leaves extract as a reducing and stabilizing agent to uphold the green chemistry principles such as less hazardous chemical synthesis. Several tools have been used to confirm the successful synthesis of the composite such as SEM, EDX, XPS, XRD, FTIR, and zeta potential which indicated the successful fabrication of the composite. The novel composite was compared with pristine nZVI for the removal aptitude of a doxycycline antibiotic with different initial concentrations to study the synergistic effect between rGO and nZVI. The adsorptive removal of bare nZVI was 90% using the removal conditions of 25 mg L^−1^, 25 °C, and 0.05 g, whereas the adsorptive removal of doxycycline by the rGO/nZVI composite reached 94.6% confirming the synergistic effect between nZVI and rGO. The adsorption process followed the pseudo-second order and was well-fitted to Freundlich models with a maximum adsorption capacity of 31.61 mg g^−1^ at 25 °C and pH 7. A plausible mechanism for the removal of DC was suggested. Besides, the reusability of the rGO/nZVI composite was confirmed by having an efficacy of 60% after six successive cycles of regeneration.

## Introduction

Water scarcity and pollution are now serious threats to all nations. Pollution of water, specifically, with antibiotics has increased in recent years because of the increased production and consumption during the COVID-19 pandemic^1–3^. Thus, developing an effective technique to eliminate antibiotics from wastewater is of great concern.

One of the resistant semisynthetic antibiotics that is derived from the tetracycline group is Doxycycline (DC)^4,5^. The remains of DC have been reported in ground and surface water as it cannot be metabolized, only 20–50% is metabolized, and the remaining is discharged into the environment causing serious environmental and health problems^6^.

Low levels contact of with DC can kill aquatic photosynthetic microbes, pose a threat to the transmission of antimicrobial bacteria, and increase antimicrobial resistance; therefore, it is a necessity to remove this pollutant from the water effluent. The natural degradation of DC in water is a very slow process. Physio-chemical processes, such as photolysis, biodegradation, and adsorption can degrade only in small concentrations and at a very slow rate^7,8^. However, adsorption is the most cost-effective, simple, environmentally friendly, facile in handling, and highly efficient approach^9,10^.

Nano-zero valent iron (nZVI) is a very effective material in the elimination of many antibiotics from water including metronidazole diazepam, ciprofloxacin, chloramphenicol, and tetracycline. This ability comes from the astonishing properties that nZVI possesses such as great reactivity, large surface area, and numerous exterior binding sites^11^. However, nZVI tends to agglomerate in aqueous media because of the Van der Wales forces and high magnetism, which reduce its efficiency in removing pollutants due to the creation of an oxide layer that inhibits nZVI reactivity^10,12^. Agglomeration of nZVI particles can be reduced by modifying their surface with surfactants and polymers or combining it with other nanomaterials in composites form which were found to be a viable method for improving its stability in the ambient medium^13,14^.

Graphene is a two-dimensional carbonaceous nanomaterial made up of sp^2^ hybridized carbon atoms arranged in a honeycomb lattice. It has a large surface area, substantial mechanical strength, excellent electrocatalytic activity, high thermal conductivity, rapid electron mobility, and an appropriate supportive material for loading inorganic nanoparticles on its surface. The combination of metal nanoparticles with graphene can substantially surpass the individual advantages of each material and allow optimal nanoparticle dispersal for more efficient water treatment due to its exceptional physicochemical properties^15^.

Plant extracts are a much better alternative for harmful chemical reducing agents that are usually used in the synthesis of reduced graphene oxide (rGO) and nZVI because they are available, inexpensive, a one-step approach, environmentally friendly, and act as a reducing and stabilizing agent at the same time because of the presence of chemicals like tannins, flavonoids, and phenolic compounds. Therefore, *Atriplex halimus* L. leaves extract was used in this study as a reducing and capping agent to synthesize the rGO/nZVI composite. *Atriplex halimus*, from the family *Amaranthaceae*, is a nitrophilous perennial shrub that extends along a wide geographic range^16^.

According to the available literature, the *Atriplex halimus* (*A. halimus*) was used for the first time for the fabrication of rGO/ nZVI composite as a cost-effective and green synthesis procedure. Consequently, the aim of this work is fourfold: (1) the phytosynthesis of rGO/nZVI composite and pristine nZVI using *A. halimus* aqueous leaves extract, (2) the characterization of the phytosynthesized composite using several techniques to confirm the successful fabrication of it, (3) to study the synergistic effect between rGO and nZVI in the adsorption removal of Doxycycline antibiotic organic pollutant using different reaction parameters, for the sake of optimizing the adsorption process conditions, (3) to examine the recyclability of the composite after different successive cycles.

## Experimental

### Materials

Doxycycline hydrochloride (DC, MW = 480.90, chemical formula C_22_H_24_N_2_O·HCl, 98%), ferric chloride hexahydrate (FeCl_3_^.^6H_2_O, 97%,), graphite powder, and were purchased from Sigma-Aldrich, USA. Sodium hydroxide (NaOH, 97%), ethanol (C_2_H_5_OH, 99.9%), and hydrochloric acid (HCl, 37%) were purchased from Merck, USA. NaCl, KCl, CaCl_2_, MnCl_2_, and MgCl_2_ were purchased from Municipality Kemi'ou Chemical Reagent Co., Ltd., Tianjin. All reagents were of high analytical grade. Double-distilled water was used for the preparation of all aqueous solutions.

### Plant extract preparation

*A. halimus* representative samples were collected from their natural habitats in the Nile Delta and the Mediterranean coastal land of Egypt. Plant material was collected following applicable national and international guidelines^17^. Professor Manal Fawzy has identified the plant specimens according to Boulos^18^ and the Department of Environmental Sciences at Alexandria University permitted the collection of the studied plant species for scientific purposes. Voucher specimens were deposited in Tanta University Herbarium (TANE) with voucher Numbers: 14,122–14,127, which is a public herbarium providing access to the deposited material. Moreover, to get rid of any dust or dirt the plant leaves were shredded into small pieces and washed three times using tap water followed by distilled water, and then oven-dried at 50 °C. The plant was ground and a fine powder amount of 5 g was immersed in 100 mL distilled water and stirred for 20 min to 70 °C to obtain the extract. The obtained *A. halimus* extract was filtered with Whatman filter paper and stored in a clean and sterilized tube at 4 °C for further use.

### Phytosynthesis of rGO/nZVI composite

**Figure 1:**
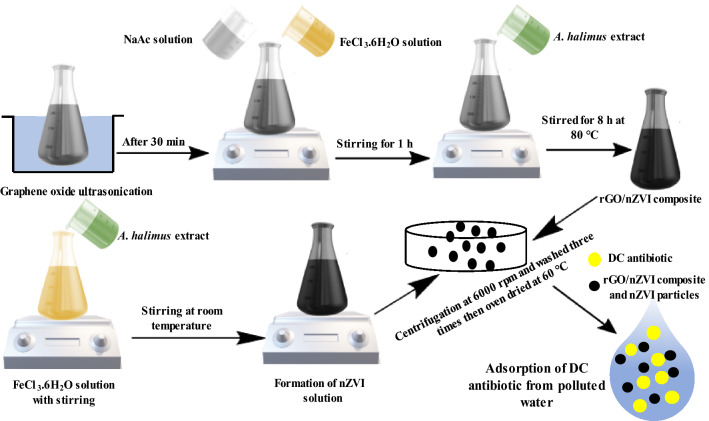
Schematic diagram and digital photos for the green synthesis of rGO/nZVI composite and nZVI using *Atriplex halimus* extract and removal of DC antibiotic from polluted water.

As illustrated in Fig. 1, GO has been fabricated from graphite powder by a modified Hummers method^19^. 10 mg of GO powder was dispersed in 50 mL deionized water under ultrasonic conditions for 30 min then 0.9 g of FeCl_3_ and 2.9 g NaAc were mixed for 60 min. 20 mL of the *Atriplex* leaves extract was added to the mixed solution at 80 °C for 8 h under stirring conditions. The obtained black suspension was filtered. Ethanol and double distilled water were used to wash the prepared nanocomposite then dried in a vacuum oven at 50 °C for 12 h.

### Phytosynthesis of nZVI

Briefly, as represented in Fig. 1, 10 mL of ferric chloride solution that contains Fe^3+^ ions with a concentration of 0.05 M was dropwise added to a 20 mL solution of *A. halimus* leaves extract under gentle heating and stirring conditions for 60 min, and then the solution was centrifugated at 14,000 rpm (Hermle, 15,000 rpm) for 15 min to get the black pellets which then washed three times using ethanol and distilled water and then dried in a vacuum oven at 60 °C overnight.

### Characterization of rGO/nZVI composite and nZVI

The phytosynthesized rGO/nZVI composite and nZVI were characterized using the UV–Visible spectroscopy (T70/T80 series UV/Vis Spectrophotometer, PG Instruments Ltd, UK), in the scanning range of 200–800 nm. TEM spectroscopy (JOEL, JEM-2100F, Japan, accelerating voltage of 200 kV) was used to analyze the morphological analysis and size distribution of rGO/nZVI composite and nZVI. To assess the possible involvement of functional groups in the plant extract that was responsible for the reduction and stabilization process, an FT-IR spectrum was conducted (JASCO spectrometer over the range 4000–600 cm^−1^). Furthermore, a zeta potential analyzer (Zetasizer Nano ZS Malvern) was used to examine the surface charge of the synthesized nanomaterial. X-ray diffractometer (X'PERT PRO. Netherland) was used for XRD measurements of powdered nanomaterials it was operated at a current of (40 mA), a voltage of (45 kV) in the 2θ range from 20° to 80° and with CuKa1 radiation ($\lambda =$ 1.54056A^o^). The energy-dispersive X-ray spectroscopy (EDX) (JEOL model JSM-IT100) was responsible to investigate the elemental composition while XPS with monochromatic X-ray Al K-alpha radiation -10 to 1350 eV spot size 400 micro m was collected on K-ALPHA (Thermo Fisher Scientific, USA) with full-spectrum pass energy 200 eV and at narrow-spectrum 50 eV. The powder sample was pressed onto a sample holder, which was placed in a vacuum chamber. C 1 s spectrum was used as a reference at 284.58 eV to determine the binding energy.

### Adsorption experiment

Adsorption experiments were performed to test the efficacy of the synthesized rGO/nZVI nanocomposite for the removal of Doxycycline (DC) from an aqueous solution. The adsorption experiment was conducted in a 25 mL conical flask with a shaking speed of 200 rpm using an orbital shaker (Stuart, orbital shaker/ SSL1) at 298 K. The desired DC working solutions (25–100 ppm) were achieved by diluting the DC stock solution (1000 ppm) with double-distilled water. For evaluating the effect of rGO/nZVI dosage on the adsorption efficiency different weights of the nanocomposite (0.01–0.07 g) were added to a 20 mL DC solution. To study the adsorption kinetic and isotherm 0.05 g of adsorbent was immersed in DC aqueous solutions with initial concentrations (25–100 mg L^−1^). The pH effect on the removal of DC has been examined at an initial concentration of 50 mg L^−1^ at pH (3–11) and 25 °C. The pH of the system was adjusted by adding small volumes of HCl or NaOH solutions (Crison pH meter, pH meter, pH 25). Also, the impact of reaction temperature on the adsorption experiment was studied in the range of 25–55 °C. The effect of ionic strength on the adsorption process was investigated by the addition of different concentrations of NaCl (0.01–4 mol L^−1^) at DC initial concentration 50 mg L^−1^, pH 3 and 7), 25 °C, and adsorbent dose 0.05 g. The adsorption of un-adsorbed DC was measured by a double beam UV–visible spectrophotometer (T70/T80 series, PG Instruments Ltd, UK) equipped with a quartz cell of 1.0 cm path length at a maximum wavelength (λ_max_) of 270 and 350 nm. The removal percentage of DC antibiotic (*R*%; Eq. 1), and the amount DC adsorbed, *q*_*t*_, Eq. 2 (mg g^−1^) was measured using the following equations^20^.

1 $$\mathrm{\%}R =\frac{{C}_{o}-C}{{C}_{o}}\times 100$$

where *%R* is DC removal aptitude (%), *C*_*o*_ is the initial concentration of DC at time zero, and *C* is the concentration of DC at time *t*, respectively (mg L^−1^).

2 $$qe =\frac{{(C}_{o}-{C}_{e})\times V}{m}$$

where *q*_*e*_ is the amount of DC adsorbed per unit mass of adsorbent (mg g^−1^), *C*_*o*_ and *C*_*e*_ are the concentrations at time zero and equilibrium, respectively (mg L^−1^), *V* is the solution volume (*L*), and *m* is the mass of adsorbent (g).

## Result and discussion

### Characterization of rGO/nZVI composite and nZVI particles

#### SEM

**Figure 2:**
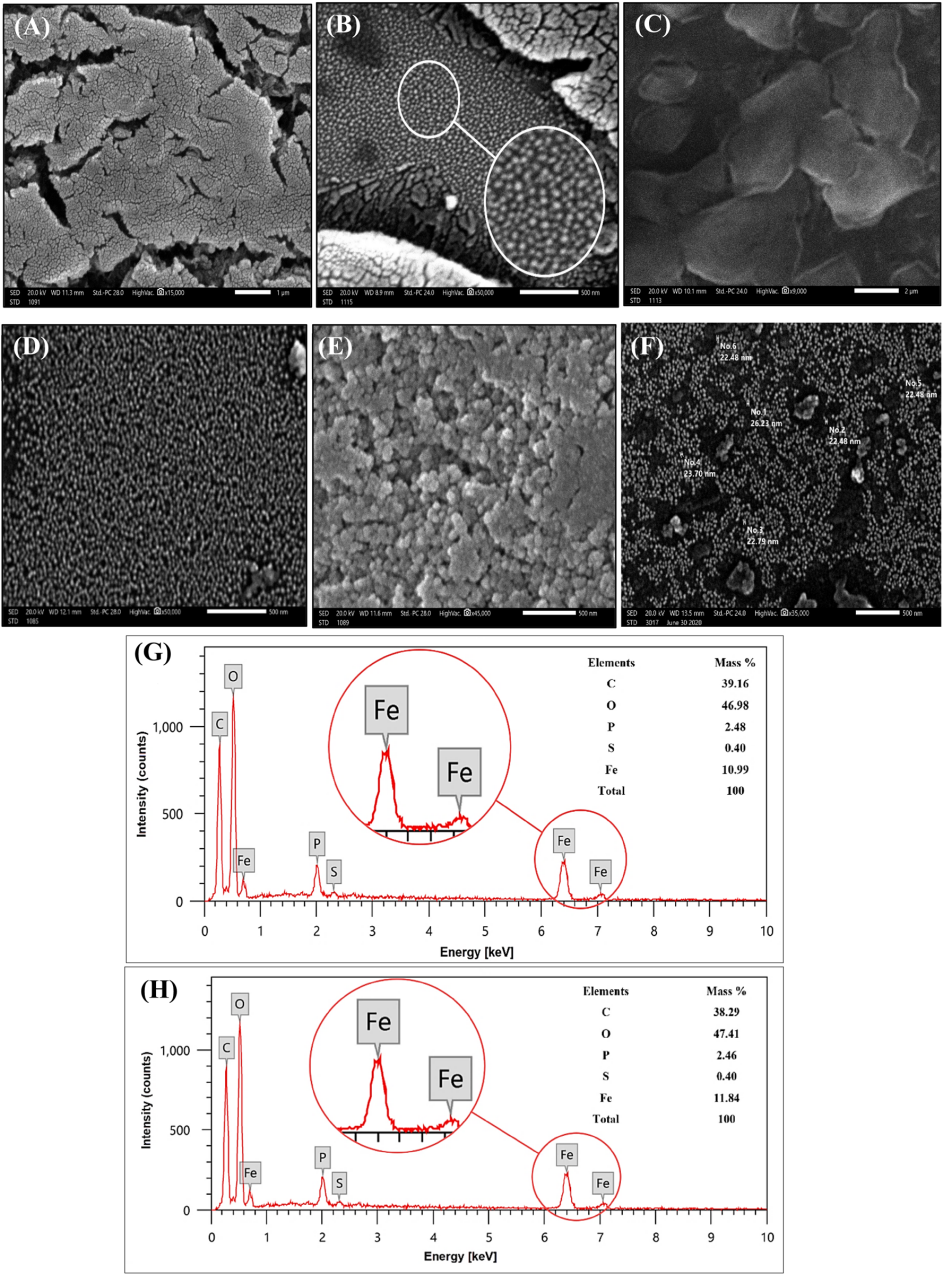
SEM images of rGO/nZVI composite (**A**–**C**), nZVI (**D**,**E**), and EDX pattern of nZVI/rGO composite (**G**), and nZVI (**H**).

The SEM images (Fig. 2A–C) depicted the sheet-like morphology of the rGO/nZVI composite with spherical iron nanoparticles dispersed uniformly on its surface thus indicating the successful attachment of nZVI NPs on the rGO surface. Moreover, the removal of oxygen-containing groups while the reduction of GO by *A. halimus* was confirmed by the presence of some wrinkles in the rGO sheets^21^. These large wrinkles act as active loading sites for the iron NPs. nZVI images (Fig. 2D–F) indicated a very well dispersion of iron NPs of spherical shape with no aggregation which is due to the capping nature of the phytoconstituents in the plant extract. The size of the particles ranged from 15–26 nm. However, some areas showed a mesoporous morphology with bumps and cavities structure that may give the nZVI high effective adsorption capacity as they can increase the possibility of trapping the DC molecules on the nZVI surface^22^. When *Rosa damascene* extracts were utilized in the synthesis of nZVI the acquired NPs were non-uniform with void space and different shapes which decreased its effectiveness in Cr (VI) adsorption and extends the reaction time^23^. The results are in line with nZVI synthesized by oak and mulberry leaves that were mostly globular nanoparticles with different nano sizes and no significant agglomeration.

#### EDX

The elemental composition of the phytosynthesized rGO/nZVI composite and nZVI was investigated through EDX (Fig. 2G,H). The investigation showed that nZVI was composed of carbon (38.29% mass), oxygen (47.41% mass), and iron (11.84% mass) however, there was a presence of other elements such as phosphorous that may come from the plant extract^24^. In addition, the high percentage of carbon content and oxygen is due to the phytochemical compounds of the plant extract present in the subsurface sample of nZVI. These elements were dispersed evenly on rGO but with different ratios, C (39.16% mass), O (46.98% mass), and Fe (10.99% mass), the EDX of rGO/nZVI also showed the presence of other elements such as S that may be due to the use of plant extract. The C:O ratio and the iron content in the current rGO/nZVI composite using *A. halimus* was much better than using *eucalyptus* leaf extract as it depicted a composition of C (23.44 wt%), O (68.29 wt%), and Fe (8.27 wt%)^25^. Nataša et al., 2022 reported similar elemental composition of nZVI synthesized by oak and mulberry leaves and confirmed that the polyphenol groups and other containing molecules in the leaf extracts are responsible for the reduction process.

#### TEM analysis

The morphology of the phytosynthesized nZVI (Fig. S2A,B) exhibits a spherical shape along with some irregular ones with an average particle size of 23.09 ± 3.54 nm; however, a chain-like aggregate was observed due to the Van der Waals forces and iron magnetic nature. This mostly granular and spherical particle shape is in good agreement with SEM results. Similar observations were detected by Abdelfatah et al., 2021 when *Ricinus communis* leaves extract was implemented in the synthesis of nZVI^11^. *Ruelas tuberosa* leaf extract was used as a reducing agent in nZVI NPs were also spherical with diameters ranging from 20 to 40 nm^26^.

The hybrid rGO/nZVI composite TEM images (Fig. S2C–D) revealed that rGO was the basal plane with edge folds and wrinkles that provided abundant loading sites for nZVI NPs; this sheet-like morphology also confirmed the successful fabrication of rGO. Furthermore, the nZVI NPs were spherical with particle sizes ranging from 5.32 to 27 nm and embedded in the rGO layers with nearly uniform dispersion. *Eucalyptus* leaf extract was used in the synthesis of Fe NPs/rGO; TEM results also confirmed that the wrinkles in the rGO layers improved the dispersion of iron NPs more than bare iron NPs and enhanced the reactivity of the composite^27^. Similar results were detected by Bagheri, et al.^28^ when the ultrasonication method was used to fabricate the composite and the average iron NPs was around 17.70 nm.

#### FTIR

**Figure 3:**
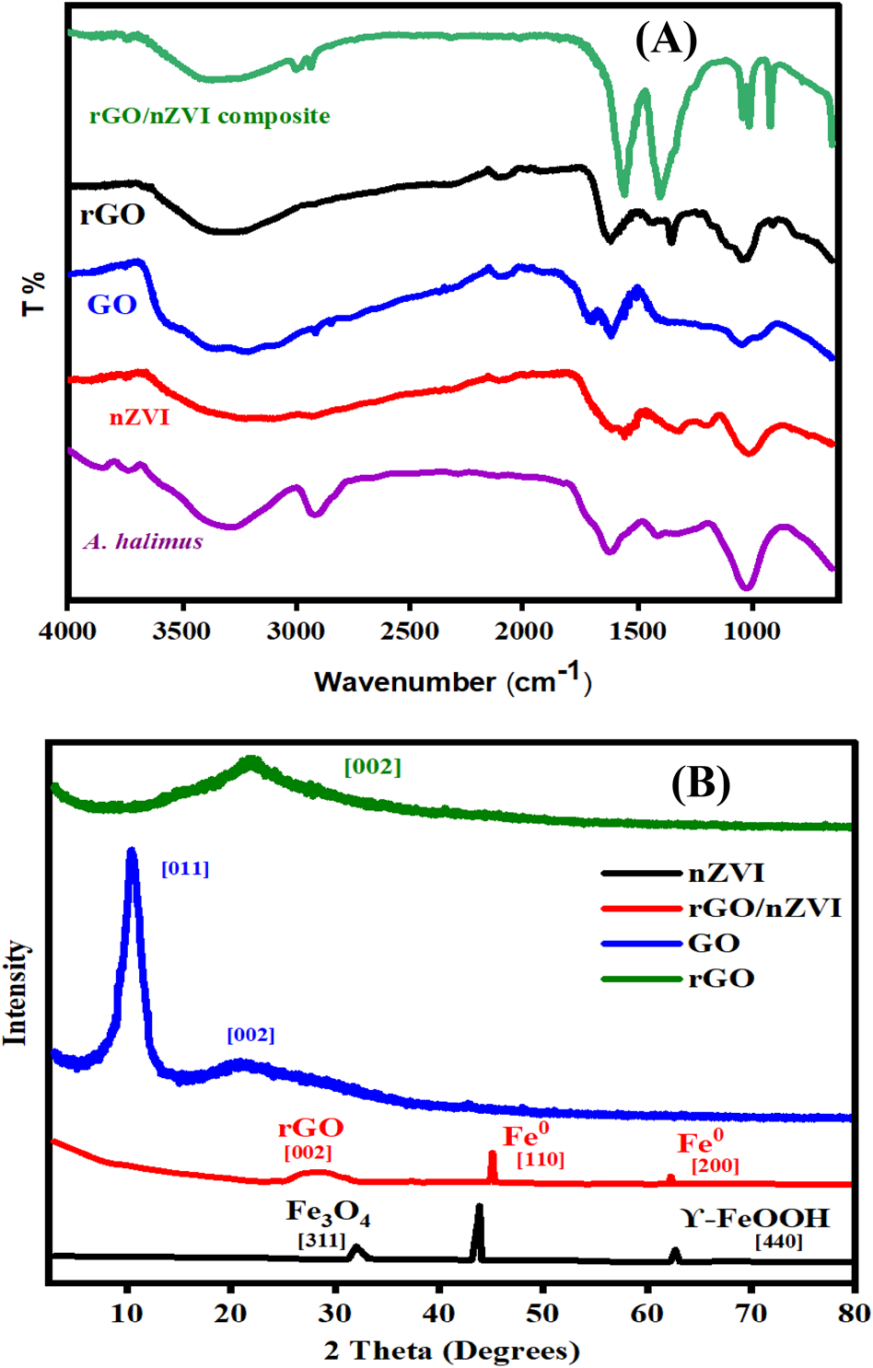
FTIR spectrum of *A. halimus*, nZVI, rGO, GO, rGO/nZVI composite (**A**). XRD pattern of rGO, GO, nZVI and rGO/nZVI composite (**B**).

The FTIR spectrum of *A. halimus*, nZVI, GO, rGO, and rGO/nZVI composite is presented in Fig. 3A. The presence of surface functional groups in the *A. halimus* leaves appeared at 3336 cm^−1^ corresponding to polyphenols and at 1244 cm^−1^ corresponding to the carbonyl group resulting from proteins. Other groups such as alkanes at 2918 cm^−1^, alkenes at 1647 cm^−1^, and CO–O–CO stretching at 1030 cm^−1^ were also observed which indicated the presence of phytoconstituents that act as capping agents and are responsible for the reduction of Fe^2+^ to Fe^0^ and GO to rGO^29^. Generally, the nZVI spectrum showed the same absorption peaks of the *A. halimus* with slight shifts in positions. A strong band appeared at 3244 cm^−1^ that is accredited to O–H stretching vibrations (phenols), the peaks at 1615 correspond to C=C, and the bands at 1546 and 1011 cm^−1^ were derived from C=O stretches (polyphenols and flavonoids), C-N group of aromatic and aliphatic amines was also observed at 1310 cm^−1^ and 1190 cm^−1^, respectively^13^. FTIR spectrum of GO depicts the existence of many oxygen-containing groups of high intensity including the alkoxy stretching band (C–O) at 1041 cm^−1^, epoxy stretching band (C–O) at 1291 cm^−1^, C=O stretching band at 1708 cm^−1^ in addition to C=C stretching band at 1619 cm^−1^, and a broad band of O–H group stretching vibration appeared at 3384 cm^−1^ which confirms the successful oxidation process of graphite by the modified Hummers method^30^. Comparing the rGO and rGO/nZVI composite to the GO spectrum, the intensity of some oxygen-containing groups such as O–H at 3270 cm^−1^ significantly deceased while others such as C=O at 1729 cm^−1^ have completely disappeared, thus indicating that oxygen-containing functional groups in the GO were successfully removed by *A. halimus* extract. New sharp rGO-characteristic peaks of C=C stretching were observed at around 1560 and 1405 cm^−1^ which confirms the reduction of GO to rGO. Shifts in 1043 to 1015 cm^−1^ and 982 to 918 cm^−1^ were observed which is likely due to the phytomaterial binding^31,32^. Weng et al., 2018 also observed significantly weakening in the oxygen-containing functional groups in the GO and that confirmed the successful formation of rGO via bioreduction as eucalyptus leaf extract that has been used to synthesize iron-reduced graphene oxide composite showed closer FTIR spectrum of phytoconstituents functional groups^33^.

#### XRD

The formation of rGO/nZVI composite and nZVI was significantly confirmed using an XRD pattern (Fig. 3B) was recorded. A high-intensity peak of Fe^0^ at 2Ɵ 44.5° was observed corresponding to indexes of (110) (JCPDS No. 06–0696)^11^. Additional peaks at 35.1° of the (311) plane were attributed to magnetite Fe_3_O_4_ and 63.2° can be indexed to the Miller indices of (440) planes due to the presence of ϒ-FeOOH (JCPDS No. 17-0536)^34^. The XRD pattern of GO indicated a sharp peak at 2Ɵ 10.3° and another peak at 21.1° pointing to the complete exfoliation of graphite and highlighting the presence of oxygen-containing groups on the surface of GO^35^. rGO and rGO/nZVI composite pattern recorded the disappearance of GO characteristic peaks and the formation of rGO broad peak at 2Ɵ 22.17 and 24.7° for rGO and rGO/nZVI composite, respectively which confirmed the successful reduction of GO by the plant extract^36^. However, additional peaks were monitored in the rGO/nZVI composite pattern at 44.9$^\circ$ and 65.22$^\circ$ related to Fe^0^ lattice planes (110), and bcc Fe^0^ (200), respectively.

#### Zeta potential

Zeta potential is the electrical potential between the layer of ions attached to the particle surface and the aqueous solution that determines the electrostatic properties of the material and measures its stability^37^. The Zeta potential analysis of the phytosynthesized nZVI, GO, and rGO/nZVI composite has revealed their stability owing to the presence of negative charges of −20.8, −22, and −27.4 mV on their surfaces, respectively, as shown in Fig. S1A–C. Such a result was in line with several reports which mentioned that solutions containing particles with zeta potential values less than −25 mV typically exhibit a high degree of stability due to the electrostatic repulsion between these particles^38^. The combination of rGO and nZVI together made the composite acquire a more negative charge and consequently a higher degree of stability than GO or nZVI alone. Thus, the electrostatic repulsion phenomenon will result in the formation of a stable rGO/nZVI composite^39^. The negative surface of GO caused its even dispersion in the water medium with no agglomeration which created a favorable condition for the interaction with nZVI. The negative charges may be attributed to the existence of various functional groups of the *A. halimus* extract which also confirms the interaction between GO and iron precursor with the plant extract to form rGO and nZVI, respectively, and rGO/nZVI composite. These phytocompounds also act as capping agents as they prevent agglomeration of the produced nanoparticles and increase their stability as a result^40^.

#### XPS

**Figure 4:**
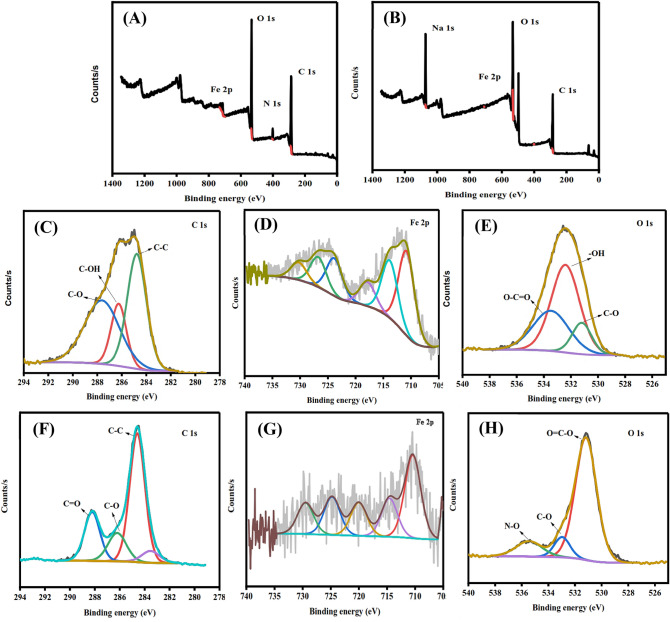
XPS survey for nZVI and rGO/nZVI composite, respectively (**A**,**B**). nZVI C1s (**C**), Fe2p (**D**), and O1s (**E**), and full spectra for rGO/nZVI composite C1s (**F**), Fe2p (**G**), O1s (**H**).

XPS technique has defined the elemental composition and the valence state of the nZVI and rGO/nZVI composite (Fig. 4). The overall XPS survey revealed that the rGO/nZVI composite is mainly composed of C, O, and Fe elements this result is in line with the EDS mapping (Fig. 4F–H). The C1s spectrum was made of three peaks at 284.59 eV, 286.21 eV, and 288.21 eV referring to C–C, C–O, and C=O, respectively. The spectrum of O1s was fitted into three peaks, including 531.17 eV, 532.97 eV, and 535.45 eV which were assigned to O=C–O, C–O, and N–O groups, respectively^41^. However, the peaks at 710.43, 714.57, and 724.79 eV are referring to Fe 2p_3/2_, Fe^+3^, and Fe p_1/2_, respectively. The XPS spectrum of nZVI (Fig. 4C–E) showed peaks of C, O, and Fe elements. The peaks at 284.77, 286.25, and 287.62 eV confirmed the existence of iron-carbon alloys as they are assigned to C–C, C–OH, and C–O, respectively. The O1s spectrum has fitted into three peaks of C–O/ferrous carbonate (531.19 eV), hydroxyl radical (532.4 eV), and O–C=O (533.47 eV). The peak at 719.6 is referring to Fe^0^ while FeOOH showed peaks at 717.3 and 723.7 eV, moreover, the peak at 725.8 eV indicated the presence of Fe_2_O_3_^42,43^.

#### BET and surface area

**Figure 5:**
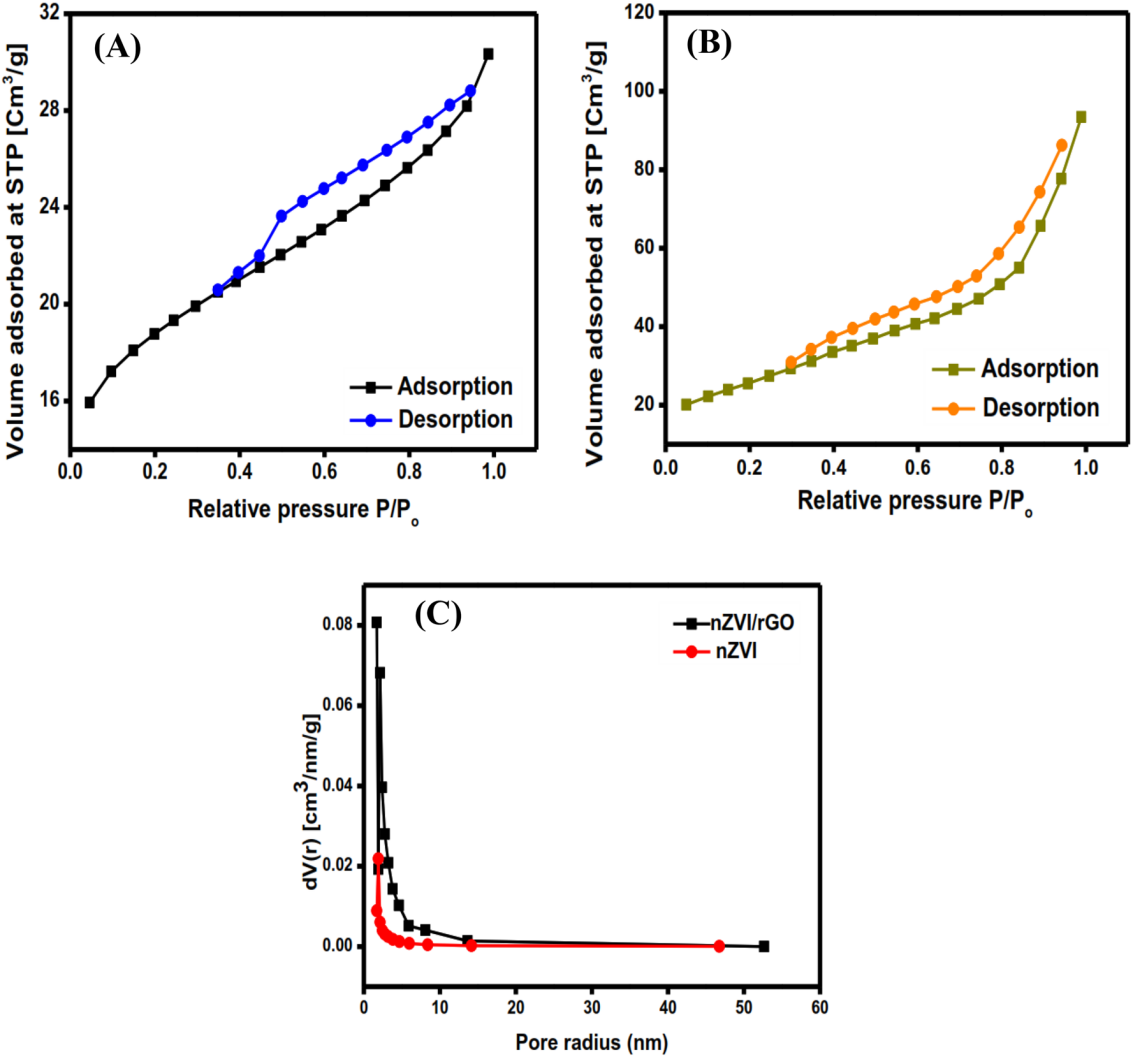
BET surface area for nZVI (**A**), rGO/nZVI composite (**B**), and pore size distribution (**C**).

It is obvious from the N_2_ adsorption/desorption isotherms (Fig. 5A,B) that nZVI and rGO/nZVI composite represent type II. Moreover, the specific surface area (S_BET_) of nZVI increased from 47.4549 to 152.52 m^2^ g^−1^ after blinding with rGO. Such a result could be explained by the decrease in the magnetism of nZVI after blinding with rGO, thereby the particle aggregation declines, increasing the surface area of the composite^44^. Also, the pore volume of the rGO/nZVI composite (8.94 nm) was higher than the pristine nZVI (2.873 nm) as represented in Fig. 5C. This result was consistent with El-Monaem, et al.^45^.

### Adsorption of DC onto rGO/nZVI composite and nZVI

**Figure 6:**
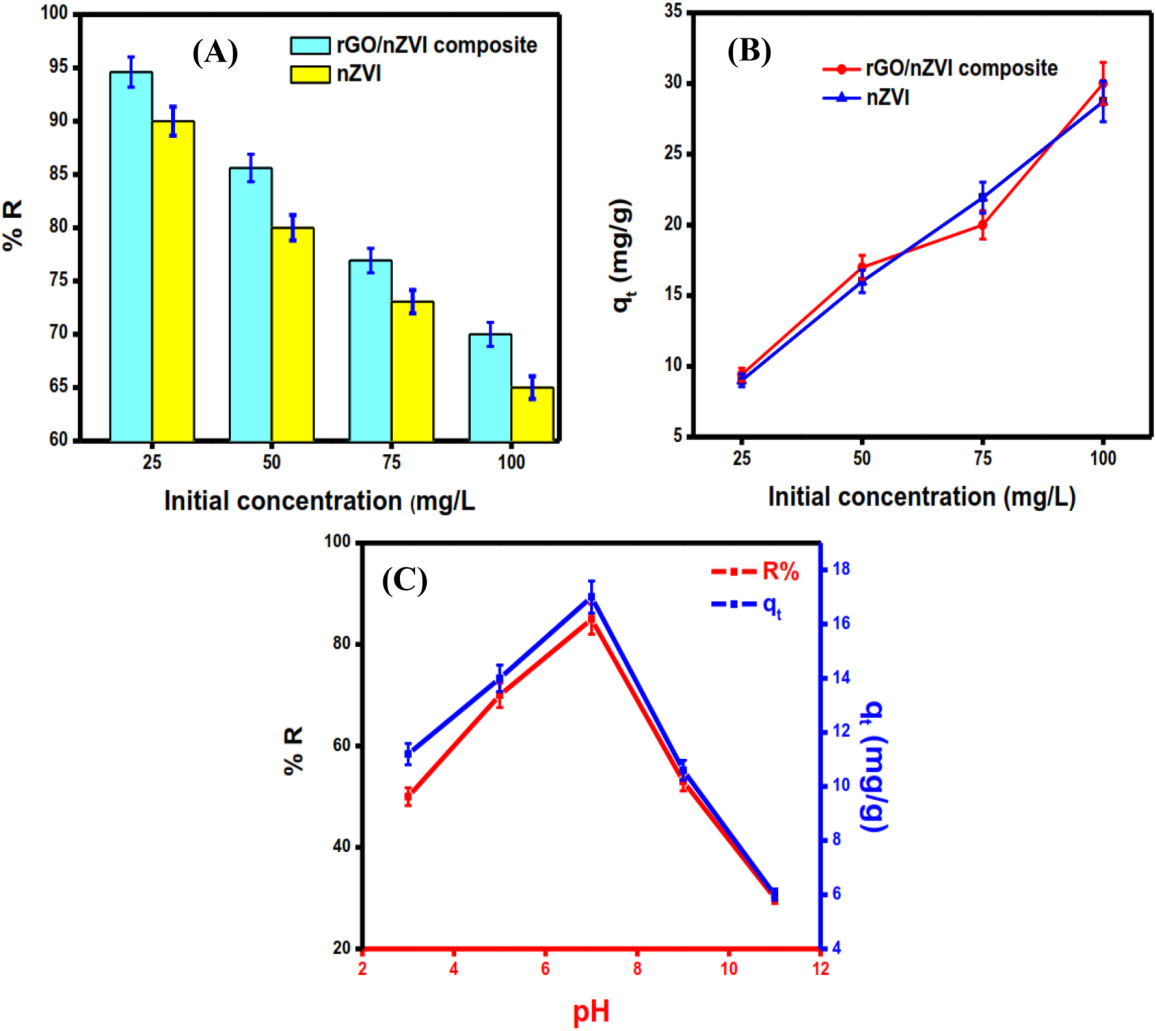
Removal efficiency and adsorption capacity of DC onto rGO/nZVI composite and nZVI, respectively (**A**,**B**) [Co = 25 mg L^−1^–100 mg L^−1^, T = 25 °C, dose = 0.05 g], pH effect on the adsorption capacity and removal efficiency of DC onto rGO/nZVI composite (**C**) [Co = 50 mg L^−1^, pH = 3–11, T = 25 °C, and dose = 0.05 g].

To evaluate the adsorption capacity between rGO/nZVI composite and pristine nZVI towards the removal aptitude of DC as a function of initial concentration increasing, a comparison study was performed by adding a constant dose of each adsorbent (0.05 g) into varied initial concentrations of DC solution [25–100 mg L^−1^] at 25 °C. The results implied that the removal efficiency of the rGO/nZVI composite (94.6%) was higher than the removal efficiency of pristine nZVI (90%) at a lower concentration (25 mg L^−1^). However, when the initial concentration increased to 100 mg L^−1^ the removal efficiency decreased to 70% and 65% in the case of rGO/nZVI and pristine nZVI, respectively (Fig. 6A), and this may be attributed to the lower active sites, and aggregation of nZVI particles. In comparison, rGO/nZVI exhibits higher DC removal efficiency, and this may be ascribed to the synergistic effect between rGO and nZVI in which the stable active sites available for adsorption were much higher and available to adsorb more DC in the case of rGO/nZVI than pristine nZVI. Furthermore, Fig. 6B reveals that when the initial concentration was increased from 25–100 mg L^−1^, the adsorption capacity of rGO/nZVI composite and nZVI increased from 9.4 mg g^−1^ to 30 mg g^−1^ and 9 mg g^−1^ to 28.73 mg g^−1^, respectively. Thus, the removal rate of DC is negatively correlated with the initial concentration of DC, which is ascribed to the limited reaction sites supported by each adsorbent for the adsorption and removal of DC in the solution. Accordingly, from these findings, it can be concluded that the rGO/nZVI composite possesses higher adsorption and reduction efficiency and that rGO in rGO/nZVI serves as both an adsorbent and supporting material.

#### pH effect

The solution pH is a crucial factor to study the adsorption process because it can affect the degree of ionization, speciation, and ionization of the sorbent. The experiment was conducted across a range of pH values (3–11) using a constant amount of adsorbent dose (0.05 g) at 25 °C, and an initial concentration of 50 mg L^−1^. According to literature survey^46^, DC is an amphoteric molecule at different pH levels with several ionizable functional groups (phenol, amino, alcohol). As a result, the various functions of DC and the related structures on the surface of the rGO/nZVI composite may interact electrostatically and may exist as cations, zwitterions, and anions, the DC molecule exists as cationic (DCH_3_^+^) at pH < 3.3, zwitterionic (DCH_2_^0^) 3.3 < pH < 7.7 and anionic (DCH^−^ or DC^2−^) at PH 7.7. The adsorption capacity and removal efficiency of DC increased from 11.2 mg g^−1^ (56%) to 17 mg g^−1^ (85%) when the pH increased from 3 to 7 (Fig. 6C). However, when the pH increased to 9 and 11 the adsorption capacity and removal efficiency decreased slightly and declines from 10.6 mg g^−1^ (53%) to 6 mg g^−1^ (30%), respectively. When the pH increased from 3 to 7 DC mainly existed as zwitterions, which made them hardly have electrostatic attraction or repulsion with rGO/nZVI composite inferring that the adsorption of DC onto rGO/nZVI composite is not dominant by electrostatic interaction. When the pH increased above 8.2 the adsorbent surface became negatively charged and thus, the adsorption capacity decreased and declined due to electrostatic repulsion between negatively charged doxycycline and the adsorbent surface. This trend revealed that DC adsorption onto the rGO/nZVI composite was strongly dependent on pH values, and the results also indicated that the rGO/nZVI composite is suitable as an adsorbent under acidic to neutral conditions.

#### Temperature effect

**Figure 7:**
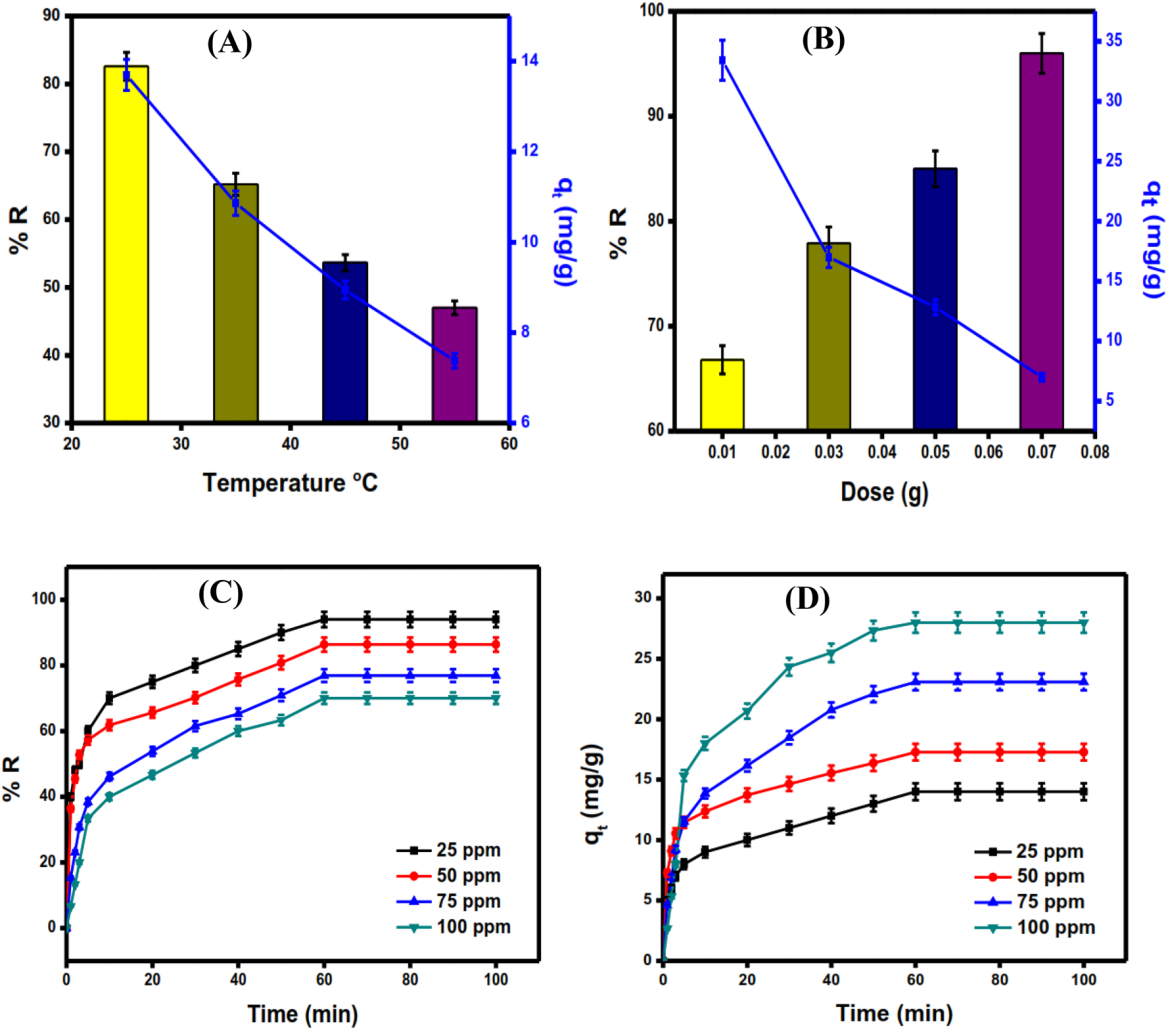
Temperature effect on removal efficiency and adsorption capacity of DC onto rGO/nZVI composite (**A**) [C_o_ = 50 mg L^−1^, pH = 7, and dose = 0.05 g], Adsorbent dose effect on the removal efficiency and removal efficiency of DC onto rGO/nZVI composite (**B**) [C_o_ = 50 mg L^−1^, pH = 7, and T = 25 °C], initial concentration effect on the adsorption capacity and removal efficiency of DC onto rGO/nZVI (**C**,**D**) [C_o_ = 25–100 mg L^−1^, pH = 7, T = 25 °C, and dose = 0.05 g].

The effect of temperature on the adsorptions of DC aqueous solution was performed at (25 °C–55 °C). The effect of increasing temperature on the removal efficiency of DC antibiotic adsorption onto rGO/nZVI was depicted in Fig. 7A, it’s clear that when increasing temperature from 25 °C to 55 °C the removal aptitude and adsorption capacity decreased dramatically from 83.44% and 13.9 mg g^−1^ to 47% and 7.83 mg g^−1^, respectively. This significant decrease could be related to an increase in the thermal energy of the DC ions, which leads to desorption^47^.

#### Adsorbent dose effect

The effect of increasing rGO/nZVI composite adsorbent dose from 0.01 g to 0.07 g on the removal efficiency and adsorption capacity is shown in Fig. 7B. The increase in adsorbent dose resulted in a drop in adsorption capacity from 33.43 mg g^−1^ to 6.74 mg g^−1^. However the removal efficiency increased from 66.8% to 96% with the increase in the adsorbent dose from 0.01 g to 0.07 g, respectively this may be attributed to the increase in the active sites on the surface of the nanocomposite^48^.

#### Initial concentration effect

The effect of initial concentration on the adsorption capacity and removal efficiency was investigated [25–100 mg L^−1^, 25 °C, pH 7, and dose 0.05 g]. When the initial concentration increased from 25 mg L^−1^ to 100 mg L^−1^, the removal percentage of the rGO/nZVI composite decreased from 94.6% to 65% (Fig. 7C), possibly due to a lack of active sites required for the adsorption of large concentrations of DC^49^. On the other hand, when the initial concentration increases the adsorption capacity also increased from 9.4 mg g^−1^ to 30 mg g^−1^ until it reaches equilibrium (Fig. 7D). This inevitable response is due to an increase in driving force with increasing initial DC concentration, which surpasses DC ion mass transfer resistance to reach the rGO/nZVI composite surface^50^.

#### Adsorption kinetics and effect of contact time

The study of contact time and kinetics aims to know the adsorption equilibrium time. Firstly, the amount of DC adsorbed during the first 40 min of contact time was about half of the total amount adsorbed during the entire time (100 min). Although DC molecules in the solution collide, causing them to migrate fast to the surface of the rGO/nZVI composite, resulting in significant adsorption. After 40 min, DC adsorption increased gradually and slowly until equilibrium was achieved at 60 min (Fig. 7D). Because a reasonable amount was adsorbed within the first 40 min, the collision of DC molecules would have been diminished, and there would have been fewer active sites available for the non-adsorbed molecules. As a result, the rate of adsorption may be slowed^51^.

**Figure 8:**
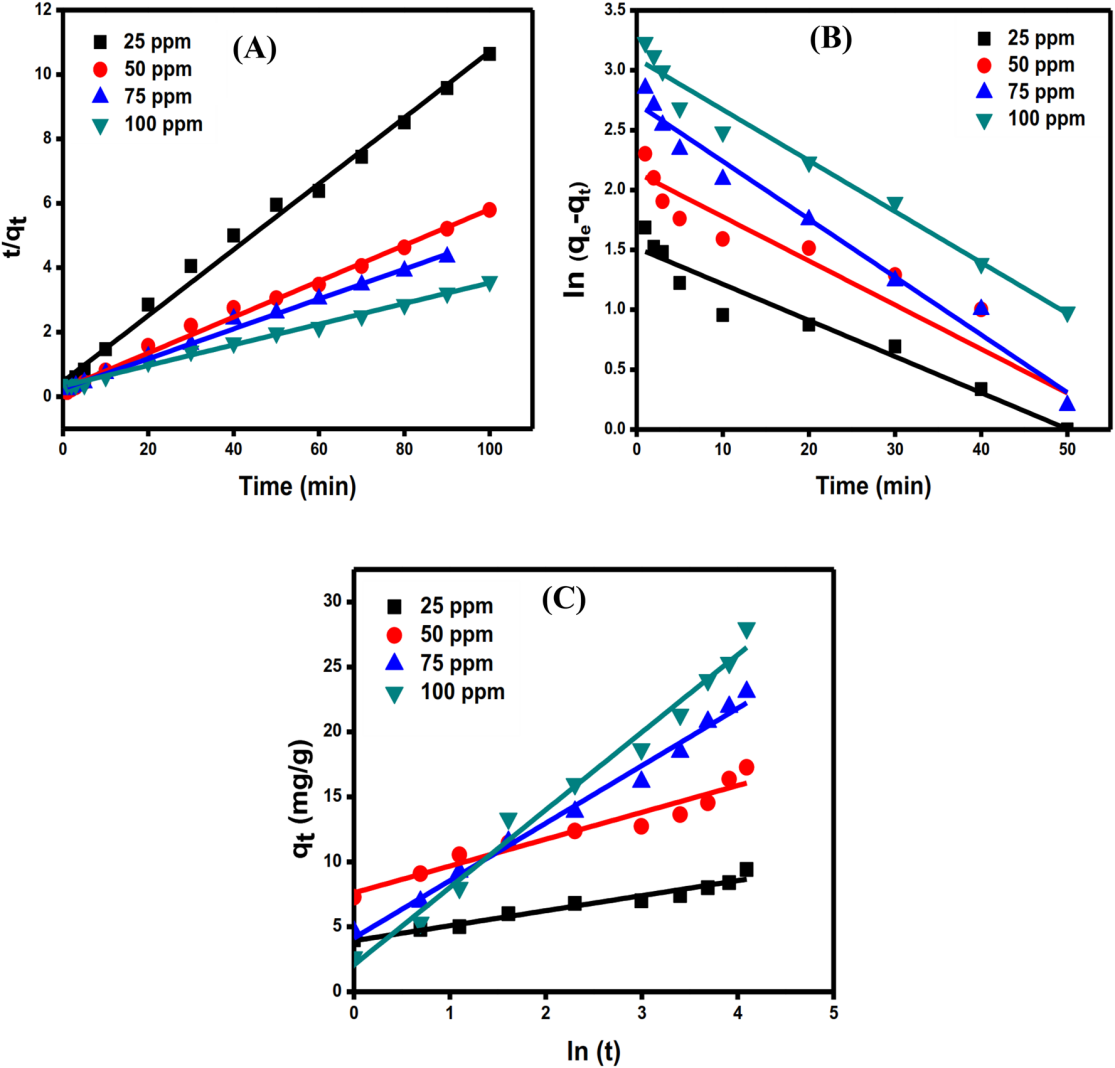
linear adsorption kinetic plots of pseudo 2nd order (**A**), pseudo 1st order (**B**), and Elovich (**C**) [C_o_ = 25–100 mg L^−1^, pH = 7, T = 25 °C, and dose = 0.05 g].

To better understand the adsorption kinetics the linear plots of pseudo 1st order (Fig. 8A), pseudo 2nd order (Fig. 8B), and Elovich (Fig. 8C) kinetic models were used. According to the parameters obtained from the kinetics study (Table S1), it’s clear that the pseudo 2nd model is the best model to depict the adsorption kinetics in which the value of *R*^2^_adjusted_ higher than the other two models. Furthermore, the similarity between the calculated adsorption capacity (q_e, cal_)_._ values from pseudo 2nd order and the experimental values (q_e, exp.)_ are another piece of evidence demonstrating that pseudo 2nd order is the best model to use when compared to the other models. As shown in Table 1, the values of *α* (the initial adsorption rate), and *β* (the desorption constant) confirmed that the rate of adsorption is greater than the rate of desorption, implying that DC has an effective tendency to be adsorbed onto rGO/nZVI composite^52^.

**Table 1:** Parameters for kinetics study on the adsorption of DC antibiotic onto rGO/nZVI composite.

Parameter	Concentration (mg L^−1^)			
	25	50	75	100
q_e, exp_ (mg g^−1^)	9.40	17.27	23.07	28
**Pseudo 1st order study**				
q_e, cal_ (mg g^−1^)	4.55	8.54	15.90	31.17
*k*_*1*_ (min^−1^)	0.03	0.036	0.04	0.042
*R*^*2*^	0.944	0.884	0.974	0.974
**Pseudo 2nd order study**				
q_e, cal_ (mg g^−1^)	9.76	15.94	24.72	31.17
*k*_*2*_ (mg g^−1^)	0.024	0.013	0.0069	0.002
*R*^2^	0.994	0.994	0.996	0.996
**Elovich study**				
β (g mg^−1^)	0.82	0.48	0.236	0.167
*α* (mg g min^−1^)	28.53	77.69	88.28	95.23
*R*^*2*^	0.970	0.940	0.989	0.986

#### Adsorption isotherms

Adsorption isotherms study aids in determining the adsorption capacity of the adsorbent (rGO/nZVI composite) at various adsorbate concentrations (DC) and system temperatures. The maximum adsorption capacity was calculated using the Langmuir isotherm, which indicates that the adsorption is homogenous and involves the formation of a monolayer of adsorbate on the adsorbent's surface with no interaction between them^53^. The two other commonly used isotherm models were Freundlich and Temkin. Although the Freundlich model is not used to calculate adsorption capacity, it does aid in understanding the heterogeneous adsorption process and that the vacant sites on the adsorbent have distinct energies, whereas the Temkin model aids in understanding the physical and chemical nature of adsorption^54^.

**Figure 9:**
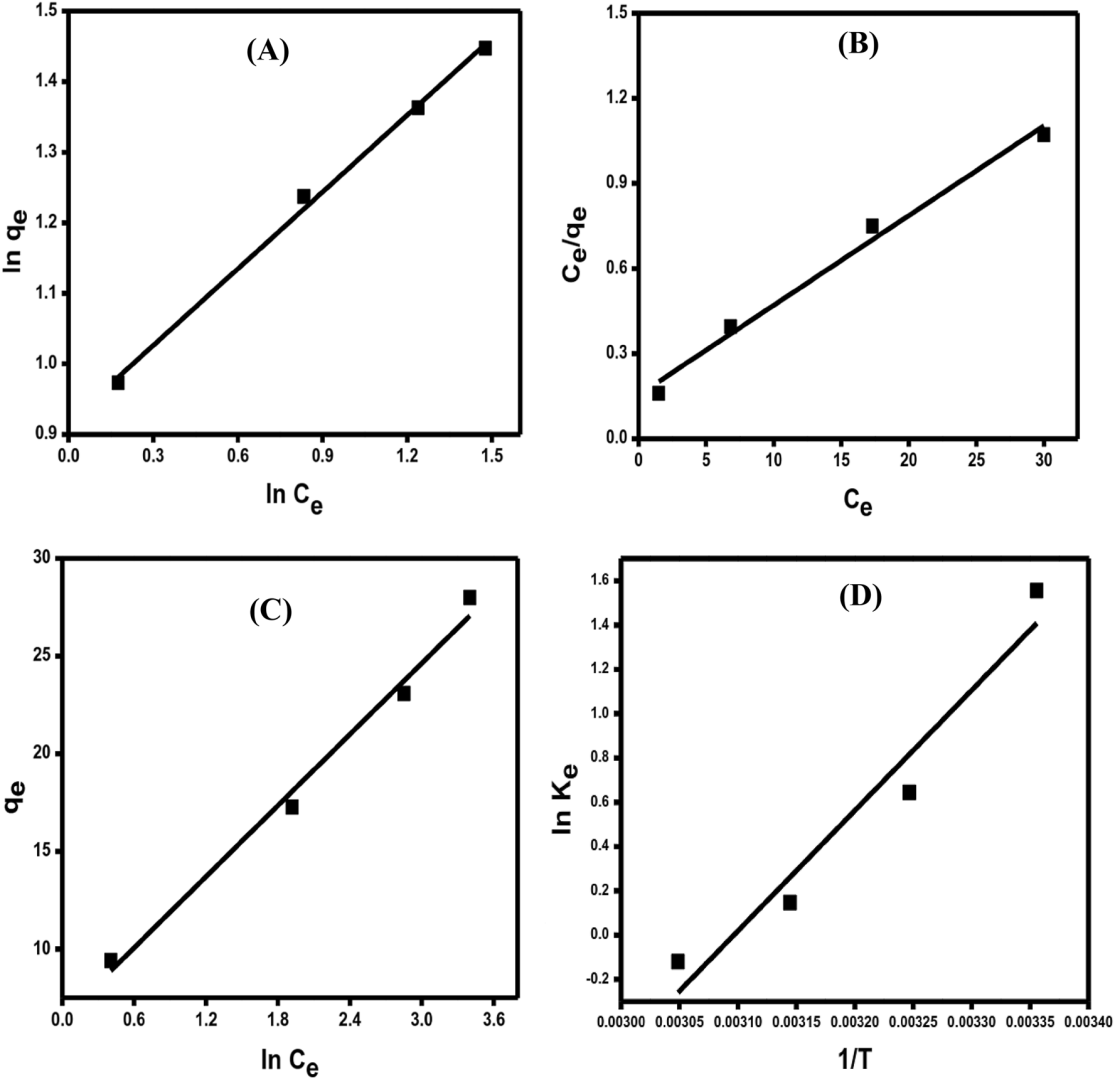
linear adsorption isotherm plots of Freundlich (**A**), Langmuir (**B**), and Temkin (**C**) [C_o_ = 25–100 mg L^−1^, pH = 7, T = 25 °C, and dose = 0.05 g]. Plot of Vant Hoff equation for the adsorption of DC onto rGO/nZVI composite (**D**) [C_o_ = 25–100 mg L^−1^, pH = 7, T = 25–55 °C, and dose = 0.05 g].

Figure 9A–C represents the linear plots of the Langmuir, Freundlich, and Temkin models, respectively. The *R*^2^ values calculated from the linear plot of Freundlich (Fig. 9A), and Langmuir (Fig. 9B) and presented in Table 2 indicated that the adsorption of DC onto the rGO/nZVI composite follows the Freundlich isotherm model (0.996) compared to Langmuir (0.988), and Temkin (0.985). The maximum adsorption capacity (q_max_) calculated from the Langmuir isotherm model was 31.61 mg g^−1^. Furthermore, the computed value of the dimensionless separation factor (*R*_*L*_) is between 0 and 1 (0.097), indicating that the adsorption process is favorable. Otherwise, the calculated Freundlich constant (*n* = 2.756) indicated the preference for this uptake process. According to Temkin isotherm linear model (Fig. 9C), the adsorption of DC onto the rGO/nZVI composite is a physical adsorption process since *b* is ˂ 82 kJ mol^−1^ (0.408)^55^. Although the physical adsorption process is generally mediated by weak Van der Waals forces, the absorption of DC onto the rGO/nZVI composite requires a low adsorption energy^56,57^.

**Table 2:** Isotherm parameters for the adsorption of DC antibiotics onto rGO/nZVI composite**.**

Isotherm model	Parameter	Value
Langmuir	[q_max_ (mg g^−1^)]	31.614
	[*K*_*L*_ (L mg^−1^)]	0.205
	(*R*_*L*_*)*	0.097
	*(R*^2^*)*	0.988
Freundlich	[*K*_*F*_ (L mg^−1^)]	2.502
	*(n)*	2.756
	(*R*^2^)	0.996
Temkin	[*B* (J mol^−1^)]	408.25
	[*b* (kJ mol^−1^)]	0.408
	*(A*)	2.882
	(*R*^2^*)*	0.985

#### Thermodynamics of adsorption

To assess the effect of change in reaction temperature on the removal of DC onto the rGO/nZVI composite, the thermodynamic parameters such as a change in entropy (ΔS), change in enthalpy (ΔH), and change in free energy (ΔG) were calculated from Eqs. 3 and 4^58^.

3 $$\mathrm{ln}{K}_{e}= \frac{\mathrm{\Delta S}}{R}-\frac{\Delta H}{RT}$$

4 $$\Delta G=\Delta H-T\Delta S$$

where ${K}_{e}$=$\frac{{C}_{Ae}}{{C}_{e}}$ is the thermodynamic equilibrium constant; C_e_ and C_Ae_ are the DC concentration in the solution onto the rGO/nZVI surface at equilibrium, respectively. R and RT are the gas constant and adsorption temperature, respectively. The plot of ln K_e_ vs. 1/T gives a straight line (Fig. 9D) from which ΔS and ΔH were determined.

The negative value of ΔH indicates that the process is exothermic. On the other hand, the value of ΔH is within the range of the physisorption process. The negative value of ΔG presented in Table 3 showed that the adsorption is feasible and spontaneous. The negative value of ΔS indicates that the molecules of the adsorbent are highly ordered at the liquid interface (Table 3).

**Table 3:** Thermodynamic parameters for DC adsorption.

ΔG (kJ/mol)				ΔH (kJ/mol)	ΔS (J/mol k)	*R*^2^
25 °C	35 °C	45 °C	55 °C	−0.49	−1.47	0.950
−0.04755	−0.03285	−0.01815	−0.00345			

#### Comparison of rGO/nZVI composite with other adsorbents

Table 4 reported a comparison between the rGO/nZVI composite and other adsorbents reported in previous studies. It is obvious that the rGO/nZVI composite has a high adsorption capacity and could be a promising material for the removal of DC antibiotics from water. Furthermore, the adsorption of the rGO/nZVI composite is a quick process with an equilibrium time of 60 min. The exceptional adsorption behavior of the rGO/nZVI composite could be attributed to the synergistic effect of rGO and nZVI.

**Table 4:** Comparison between the maximum adsorption capacities and equilibrium time of DC antibiotics removal onto different reported adsorbents with rGO/nZVI composite of the current study.

Different adsorbents	Removal efficiency (%)	Ref
Cu (II) impregnated biochar	93.22	^59^
Graphene oxide/hydrogel composite	85	^60^
Fe_3_O_4_ magnetic nanoparticles	98.7	^61^
Spent black tea leaves	83	^62^
Electro-generated adsorbents	92.8	^63^
ferrihydrite/plant-based composites	95	^64^
CuO/Fe_2_O_3_ (CF) composite	90	^62^
Magnetic Fe_3_O_4_@chitosan carbon microbeads	80	^65^
rGO/nZVI composite	94.6	This work

#### Adsorption mechanism of DC on rGO/nZVI composite and nZVI

**Figure 10:**
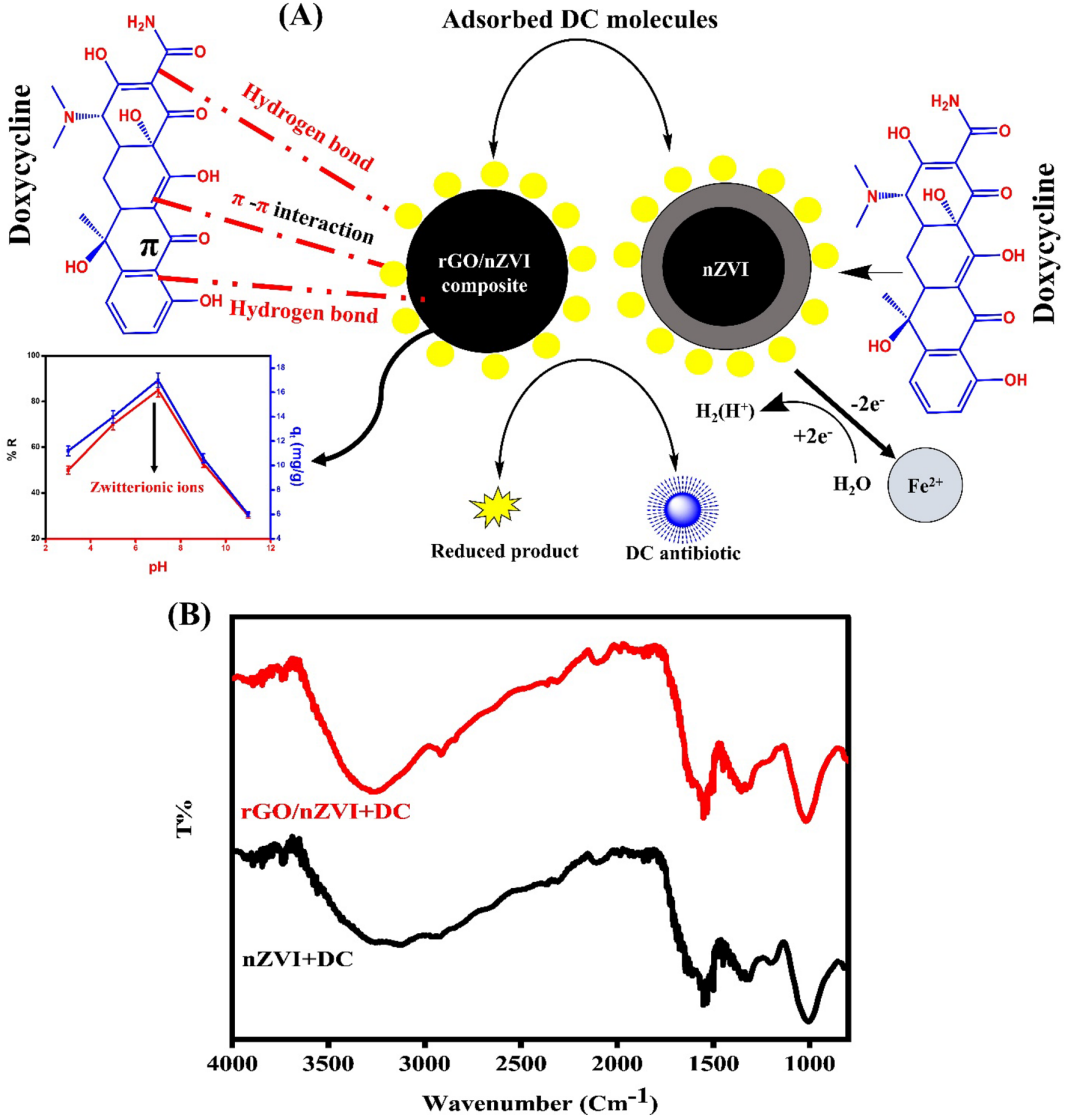
Plausible adsorption mechanism for the removal of DC antibiotic by rGO/nZVI composite and nZVI (**A**). FTIR spectra for the adsorption of DC onto rGO/nZVI and nZVI (**B**).

Figure 10A,B elucidates the plausible mechanism for the removal of DC antibiotics by rGO/nZVI composite and nZVI. According to the experimental results of the effect of the pH on the DC adsorption efficiency, the adsorption of DC onto rGO/nZVI composite is not controlled by electrostatic interaction as it acts as a zwitterionic ion when the pH increases from 3 to 7 so, the change in pH does not affect the adsorption process. Subsequently, the adsorption mechanism may be governed by non-electrostatic interactions such as hydrogen bonding, hydrophobic effect, and π − π stacking interaction between rGO/nZVI composite and DC^66^. As is well known, the mechanism of aromatic adsorbate to layered graphene surface has always been explained in terms of π − π stacking interaction as the main driving force^46^. The composite, which is a layered material like graphene has a maximum absorption at 233 nm due to the π − π* transition. Based on the presence of four aromatic rings present in the molecular structure of the adsorbate DC, we postulated a mechanism of π − π stacking interaction between aromatic compound DC (π-electron-acceptor) and π electron-rich regions on the surface of rGO/nZVI composite. Moreover, as shown in Fig. 10B FTIR study was performed to probe the molecular interaction of the rGO/nZVI composite with DC, and the FTIR spectra of the rGO/nZVI composite after DC adsorption are displayed in Fig. 10B. New peaks were observed at 2111 cm^−1^ corresponding to the skeletal vibration of C=C bonds indicating the presence of the corresponding organic functional groups on the rGO/nZVI surface ^67^. Other peaks were shifted from 1561 to 1548 cm^−1^ and 1399 to 1360 cm^−1^ which also confirmed that π − π interactions play important roles in the adsorption process between graphene and organic pollutants^68,69^. The decrease in the intensity of some oxygen-containing groups such as O–H at 3270 cm^−1^ after adsorption of DC indicates that hydrogen bonding is one of the mechanisms of adsorption^70^. So, based on the results the adsorption of DC onto the rGO/nZVI composite is mainly due to π − π staking interactions, and H-bonding.

In comparison with nZVI, after the adsorption of DC onto nZVI (Fig. 10B), nZVI absorption bands at 3244, 1615, 1546, and 1011 cm^−1^ were increased in intensity which should be related to the interaction with the possible carboxylic acid functional groups in DC. However, this lower transmission percentage at all observed bands, compared to nZVI before the adsorption process, denotes that the adsorption efficiency of phytosynthesized adsorbent (nZVI) did not significantly change^22^. According to some studies performed on the removal of DC using nZVI^71^ When nZVI and H_2_O interacted, electrons were released, and H^+^ was then used to produce active hydrogen with high reducibility. Finally, some cationic compounds accepted electrons from active hydrogen, leading to –C=N and –C=C–, which are attributed to the cleavage of the benzene ring.

#### Effect of ionic strength

**Figure 11:**
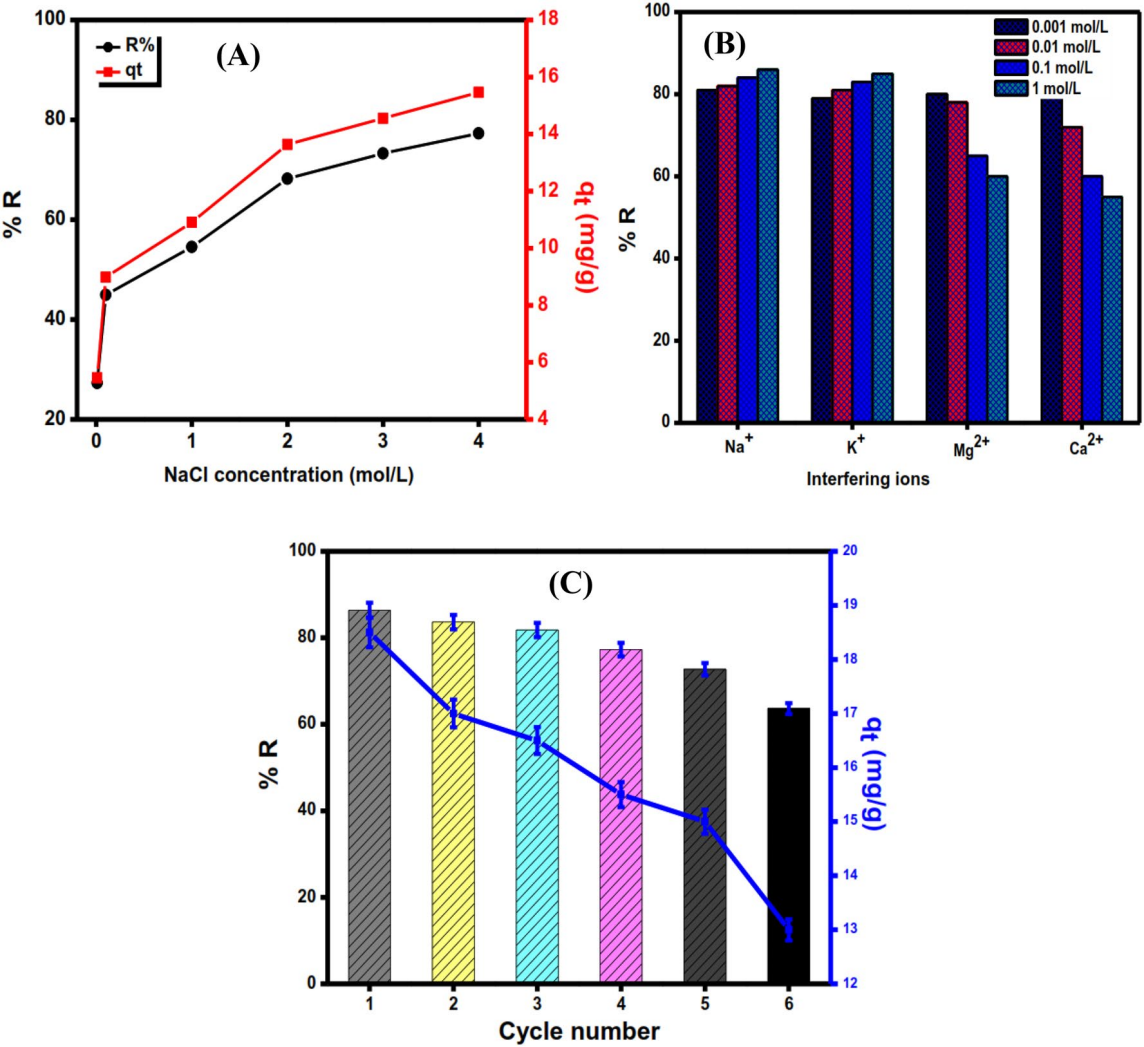
Effect of ionic strength of NaCl (**A**), and interfering ions (**B**) on the adsorption removal of DC onto rGO/nZVI composite [C_o_ = 50 mg L^−1^, pH = 7, T = 25 °C, NaCl concentration = 0.01–4 mol L^−1^, interfering ions concentration = 0.001–1 mol L^−1^, and dose = 0.05 g]. Regeneration and reusability of rGO/nZVI composite in DC adsorption (**C**) [C_o_ = 50 mg L^−1^, pH = 7, T = 25 °C, and dose = 0.05 g].

The effect of ionic strength on the DC adsorption process is shown in Fig. 11A. When the concentration of NaCl was increased from 0.01 mol L^−1^ to 4 mol L^−1^, the adsorption capacity, and removal efficiency increased gradually from 5.46 mg g^−1^ to 15.50 mg g^−1^ and 27.28% to 77.3% by increasing the concentration of NaCl from, respectively. Within the adsorption process, there are two possible effects. The salting-out effect, which is caused by an increase in ionic strength at circumneutral conditions, is one effect. The other is that the ions in the solution may infiltrate into the diffuse double layer over the surfaces of the rGO/nZVI composite materials and remove the electrostatic repulsive force between the adsorbent^72^. With increasing NaCl concentration, the uptake of DC onto the rGO/nZVI composite slightly improved, indicating that the salting out effect is predominant at different NaCl concentrations.

#### Effect of interfering ions

The effect of interfering ions may affect the adsorption efficiency of the aqueous phase. However, studying the adsorption influence of main ions such as (Na^+^, K^+^, Mg^2+^, and Ca^2+^) on DC antibiotics was essential. The study was performed by adding various concentrations of coexisting ions (0.001–1 mol L^−1^) at 50 mg L^−1^, pH 7, and 25 °C. Figure 11B showed that the adsorption efficiency was influenced by the effect of coexisting ions. Na^+^ and K^+^ influence the adsorption efficiency of DC when the concentration increases from 0.001 mol L^−1^ to 1 mol L^−1^ while the effect of Ca^2+^ and Mg^2+^ inhibits the adsorption efficiency of DC as the concentration increases. The following could be used to explain these phenomena. Since divalent cations have a stronger polarizing power and ionic strength than monovalent cations, the salting-out effect has always been greater than the squeezing-out effect^73,74^. Furthermore, stronger direct hydration and less interaction with active sites on the surface of the rGO/nZVI composite could result from the increased covalent property of divalent cations compared to monovalent cations. Consequently, the cations had a significant impact on the absorption of DC antibiotics^47^.

#### Reusability of rGO/nZVI composite

Regeneration capacity is an important indicator to evaluate the performance of adsorbent material. Six cycles of the adsorption/desorption process were performed to evaluate the renewability of the phytosynthesized rGO/nZVI composite for reuse many times for the removal of DC antibiotics. Figure 11C indicates that the removal efficiency of the phytosynthesized nanocomposite exceeds 60% after the sixth cycle with a maximum adsorption capacity of 18.5 mg g^−1^. These findings confirm the good recyclability of the phytosynthesized rGO/nZVI composite.

## Conclusion

In the current work, a facile and completely green procedure was employed for the fabrication of nZVI and a novel rGO/nZVI composite using *A. halimus* leaves extract. The characterization results of nZVI and rGO/nZVI confirmed that rGO can enhance the dispersion and stability of nZVI. The increase in the amount of nZVI being loaded in the folds of rGO resulted in a greater specific surface area and pore volume for rGO/nZVI. These structural changes facilitated the interaction between DC and rGO/nZVI. Subsequently, it increased the removal efficiency of DC. rGO/nZVI composite showed a great adsorption removal aptitude of DC antibiotics at different DC initial concentrations and optimum reaction conditions (DC concentrations: 25–100 mg L^−1^, 25 °C, pH 7, and adsorbent dose of 0.05 g). The effect of different parameters was also scrutinized by varying the pH level, temperature, adsorbent dose, contact time, and the initial concentration of adsorbate. Results showed that the optimum removal conditions were pH = 7, T = 25 °C, and dose = 0.07 g. A deep investigation of adsorption isotherm models and kinetic studies led to the conclusion that the adsorption of DC onto rGO/nZVI composite was fitted to Freundlich with a maximum adsorption capacity of 31.61 mg g^−1^ at 25 °C and followed pseudo-second order kinetic model. The result of thermodynamic studies clarified that the adsorption process could be spontaneous, feasible, and exothermic. The rGO/nZVI had an outstanding recycling capability even after six successive cycles along with superior performance for DC adsorption. Consequently, rGO/nZVI constituted a novel composite that is speculated to be effective when applied in the adsorption of other environmental pollutants.

## Supplementary Information

Supplementary Information.

## Data Availability

The data presented in this study are available on request from the corresponding author.
