# Supplementary material for: Synergetic effect of green synthesized reduced graphene oxide and nano-zero valent iron composite for the removal of doxycycline antibiotic from water
Source: Sci Rep. 2022 Nov 12;12:19372. doi: 10.1038/s41598-022-23684-x (PMC9652592; doi:10.1038/s41598-022-23684-x)
Supplement: Supplementary file 1 — Supplementary Information. [file 41598_2022_23684_MOESM1_ESM.docx]

**Supplementary data**

**Synergetic effect of green synthesized reduced graphene oxide and nano-zero valent iron composite on the removal of doxycycline antibiotic from water**

**Ahmed M. Abdelfatah^1,2, *^, Nourhan El-Maghrabi^1,2^, Alaa El Din Mahmoud^1,2^, Manal Fawzy^1,2,3^**

^1^ Environmental Sciences Department, Faculty of Science, Alexandria University, 21511 Alexandria, Egypt.

^2^ Green Technology Group, Faculty of Science, Alexandria University, 21511 Alexandria, Egypt

^3^ National Biotechnology Network of Expertise (NBNE), Academy of Scientific Research and Technology (ASRT), Egypt.
[AhmedMohamedFatah@alexu.edu.eg](mailto:AhmedMohamedFatah@alexu.edu.eg)

[nourhan.elmaghrabi@alexu.edu.eg](mailto:nourhan.elmaghrabi@alexu.edu.eg)

[alaa-mahmoud@alexu.edu.eg](mailto:alaa-mahmoud@alexu.edu.eg)

[dm_fawzy@yahoo.com](mailto:dm_fawzy@yahoo.com)

Ahmed M. Abdelfatah ORCID ID: 0000-0002-3361-8320

Nourhan EL Maghrabi ORCID ID: 0000-0001-6097-3208

Alaa El Din Mahmoud ORCID ID: 0000-0001-6530-9816

Manal Fawzy ORCID ID: 0000-0002-9401-9049

**Experimental work**

**Regeneration of rGO/nZVI adsorbent**

NaOH of 0.1 mol L^-1^ was used after the adsorption process by stirring at 200 rpm and room temperature for 60 min. Following desorption, the supernatant was disposed of, and the residual solution was oven-dried at 50 ℃ for the following adsorption experiment. In all cycles, the adsorbent amount was 0.05 g with a DC concentration of 50 mg L^-1^. The mentioned procedure was replicated 6 times to confirm material reuse efficiency.

**Result and discussions**


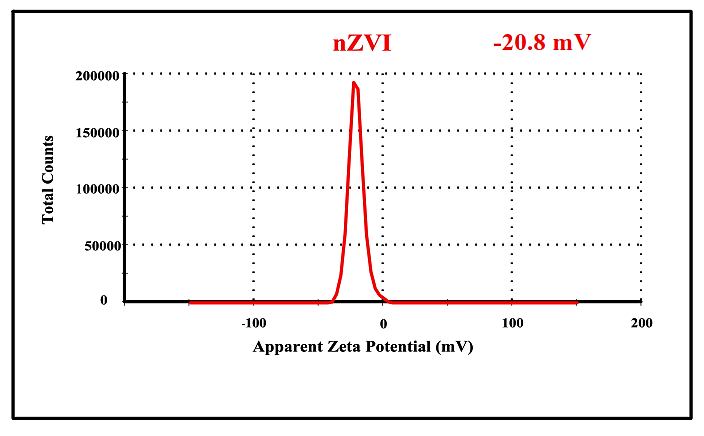

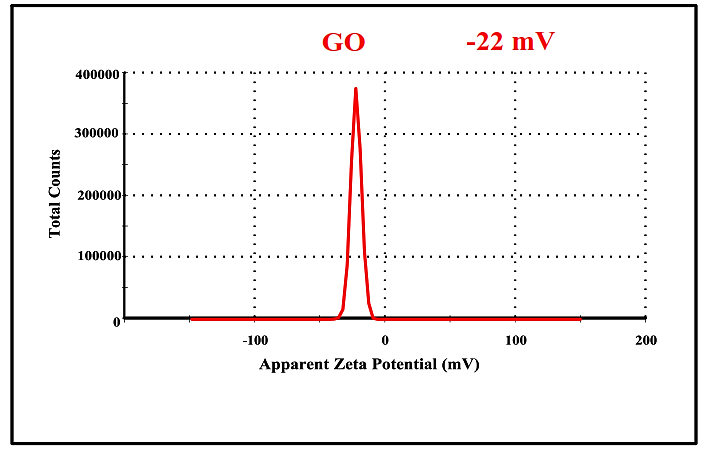


**(A)**

**(B)**


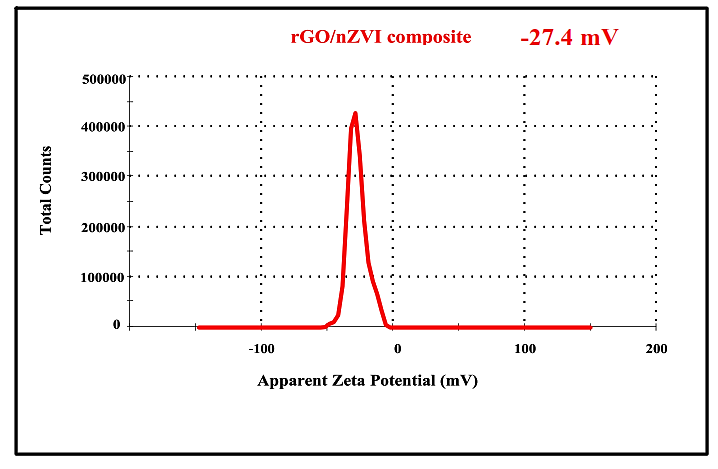


**(C)**

**Figure S1.** Zeta potential of the nZVI (A)**,** GO (B), and rGO/nZVI composite (C).

**3.1.3. TEM analysis**


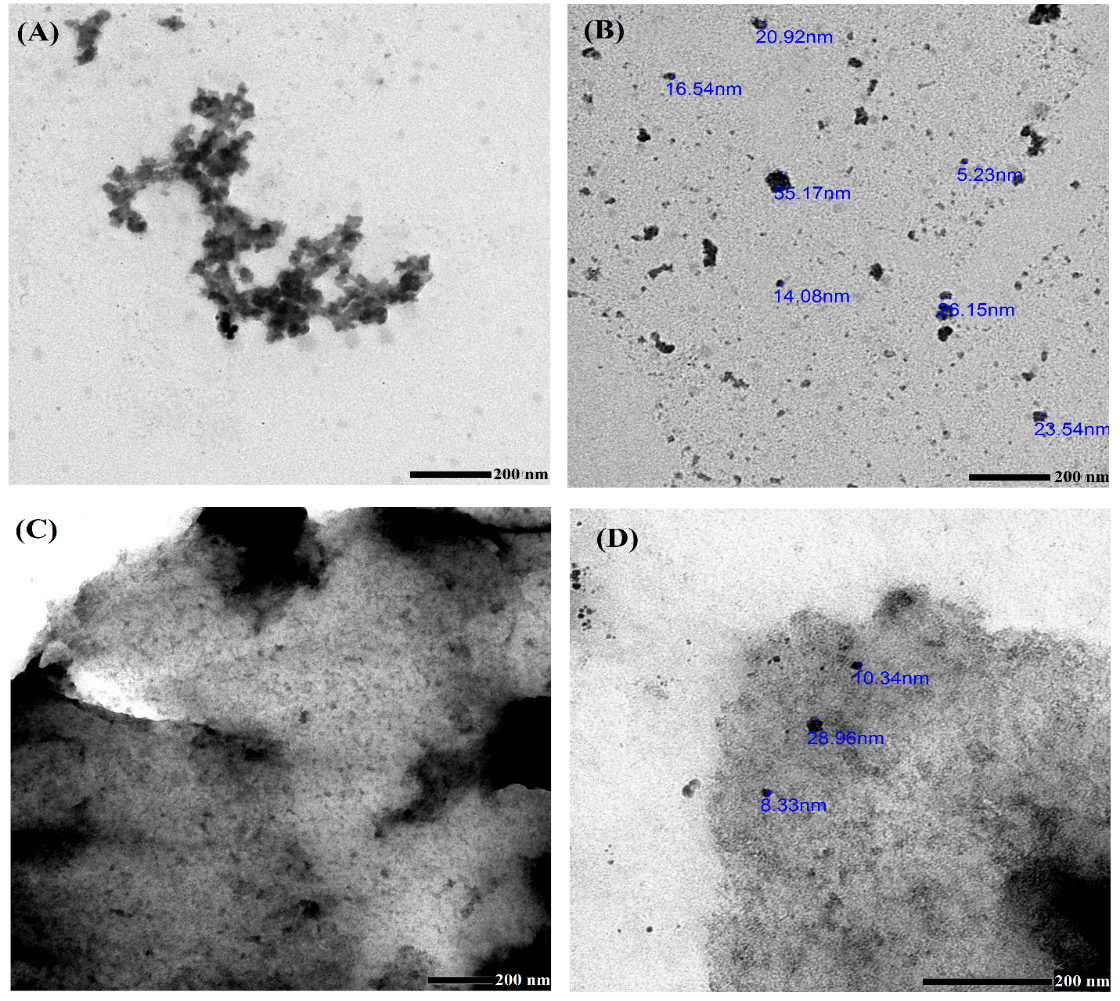


**Figure S2.** TEM images of nZVI (A), the distribution and size of nZVI particles (B), rGO-nZVI composite (C), and the distribution and size of nZVI particles onto the rGO sheets (D).

**Table S1**

Adsorption isotherm and kinetics models utilized in the current study.

|  | **Model** | **Linear form** |
| --- | --- | --- |
| **Kinetic models** | Pseudo 1^st^ order | $\ln\left( \boldsymbol{q}_{\boldsymbol{e}}\boldsymbol{-}\boldsymbol{q}_{\boldsymbol{t}} \right)\boldsymbol{=}\mathbf{ln}\boldsymbol{q}_{\boldsymbol{e}}\boldsymbol{-}\boldsymbol{K}_{\boldsymbol{1}}\boldsymbol{t}$ |
|  | Pseudo 2^nd^ order | $\frac{\mathbf{t}}{\boldsymbol{q}_{\boldsymbol{t}}}\mathbf{=}\frac{\mathbf{1}}{\boldsymbol{K}_{\boldsymbol{2}}\mathbf{x}{\boldsymbol{q}_{\boldsymbol{e}}}^{\mathbf{2}}}\mathbf{+}\frac{\boldsymbol{1}}{\boldsymbol{q}_{\boldsymbol{e}}}$ |
|  | (Elovich) | **q_t_ = [1/β ln (αβ)] + [1/β ln (t)]** |
| **Adsorption isotherm models** | Langmuir | **C_e_/q_e_ =1/K_L_ q_m_ + C_e_/q_m,_**  ***R_L_*=**$\frac{\boldsymbol{1}}{\boldsymbol{1+}\boldsymbol{K}_{\boldsymbol{l}} \boldsymbol{C}_{\boldsymbol{o}}}$ |
|  | Freundlich | $\ln\left( \boldsymbol{q}_{\boldsymbol{e}}\boldsymbol{)=}\mathbf{ln}\boldsymbol{(}\boldsymbol{K}_{\boldsymbol{f}} \right)\boldsymbol{+}\frac{\boldsymbol{1}}{\boldsymbol{n}}\mathbf{ln}\boldsymbol{(}\boldsymbol{C}_{\boldsymbol{e}}\boldsymbol{)}$ |
|  | Temkin | **q_e_ = B_1_ . log A + B_1_ log Ce** |

Where q_e_, q_t_, q_m_ is the amount of adsorbed dye at equilibrium, the amount of dye adsorbed at time t, and the maximum adsorption capacity (mg. g^−1^), respectively. *k_1_*, *k*_2_ is the rate constant of pseudo-first-order (min^-1^) and rate constant of pseudo-second-order (g mg^−1^ min^−1^). *β* is the desorption constant (g mg^-1^) and α is the initial adsorption rate (mg g^-1^ min^-1^). *C_e_* is the equilibrium concentration (mg L^−1^), *K_L_* is the adsorption equilibrium constant (L. mg^−1^), *R*_L_ is the separation factor. B_1_ is the Temkin constant, A is the equilibrium bond constant.
